# Bias of health estimates obtained from chronic disease and risk factor surveillance systems using telephone population surveys in Australia: results from a representative face-to-face survey in Australia from 2010 to 2013

**DOI:** 10.1186/s12874-016-0145-z

**Published:** 2016-04-18

**Authors:** Eleonora Dal Grande, Catherine R. Chittleborough, Stefano Campostrini, Anne W. Taylor

**Affiliations:** Population Research and Outcome Studies, Discipline of Medicine, The University of Adelaide, SAHMRI, Level 7, North Terrace, Adelaide, SA 5005 Australia; School of Public Health, The University of Adelaide, Adelaide, Australia; University Ca’ Foscari, Venice, Italy

**Keywords:** Bias, Telephone sampling methodology, Sampling frame, Public health surveillance, Health surveys, Chronic conditions, Risk factors

## Abstract

**Background:**

Emerging communication technologies have had an impact on population-based telephone surveys worldwide. Our objective was to examine the potential biases of health estimates in South Australia, a state of Australia, obtained via current landline telephone survey methodologies and to report on the impact of mobile-only household on household surveys.

**Methods:**

Data from an annual multi-stage, systematic, clustered area, face-to-face population survey, Health Omnibus Survey (approximately 3000 interviews annually), included questions about telephone ownership to assess the population that were non-contactable by current telephone sampling methods (2006 to 2013). Univariable analyses (2010 to 2013) and trend analyses were conducted for sociodemographic and health indicator variables in relation to telephone status. Relative coverage biases (RCB) of two hypothetical telephone samples was undertaken by examining the prevalence estimates of health status and health risk behaviours (2010 to 2013): directory-listed numbers, consisting mainly of landline telephone numbers and a small proportion of mobile telephone numbers; and a random digit dialling (RDD) sample of landline telephone numbers which excludes mobile-only households.

**Results:**

Telephone (landline and mobile) coverage in South Australia is very high (97 %). Mobile telephone ownership increased slightly (7.4 %), rising from 89.7 % in 2006 to 96.3 % in 2013; mobile-only households increased by 431 % over the eight year period from 5.2 % in 2006 to 27.6 % in 2013. Only half of the households have either a mobile or landline number listed in the telephone directory. There were small differences in the prevalence estimates for current asthma, arthritis, diabetes and obesity between the hypothetical telephone samples and the overall sample. However, prevalence estimate for diabetes was slightly underestimated (RCB value of −0.077) in 2013. Mixed RCB results were found for having a mental health condition for both telephone samples. Current smoking prevalence was lower for both hypothetical telephone samples in absolute differences and RCB values: −0.136 to −0.191 for RDD landline samples and −0.129 to −0.313 for directory-listed samples.

**Conclusion:**

These findings suggest landline-based sampling frames used in Australia, when appropriately weighted, produce reliable representative estimates for some health indicators but not for all. Researchers need to be aware of their limitations and potential biased estimates.

**Electronic supplementary material:**

The online version of this article (doi:10.1186/s12874-016-0145-z) contains supplementary material, which is available to authorized users.

## Background

Many established population-based, continuous chronic disease and behavioural risk factor surveillance systems worldwide utilise Computer Assisted Telephone Interviewing (CATI) [1–9]. Since the 1990s, CATI surveys have been seen as an ideal tool since they are effective, relatively inexpensive, flexible and timely [6, 8–12]. However, over the past 15 years vast changes have occurred in the telecommunication industry (mobile telephone and internet) and society’s acceptance of, and engagement with, these new technologies [13, 14]. The new communication technologies have had an impact on population-based telephone surveys, specifically, the diminishing coverage of traditional sampling frames and declining response rates [11, 15] resulting in increased costs [16, 17] and potential bias in survey estimates [18, 19].

In the early 1990s, 95–97 % of Australian households had a landline telephone connected [20] and response rates of around 70–80 % were the norm [20–24]. For population health surveys in Australia, two sampling methodologies were used: directory-listed telephone numbers, referred to as Electronic White Pages (EWP) and random digit dialling (RDD) of landline telephone numbers [3, 20, 22]; both methods having the ability to target geographical areas (state, suburbs or postcodes) which has contributed to the utility and efficiency of telephone surveys [25, 26]. EWP consists mainly of listed landline telephone numbers with name and address details for a household or business which the sampling frame can be easily stratified by state, suburb or postcode. EWP has mobile and Voice over Internet Protocol (VOIP) telephone numbers but only as a small proportion of the total sample. One drawback of EWP is that it does not include unlisted (silent) telephone number; that is, households which have opted, at a cost, to exclude their landline telephone number from the EWP. RDD methods have been developed to include silent landline telephone numbers based on the prefixes of the landline telephone numbers. Some of these methods use the EWP, known as list-assisted RDD (LA-RDD), to make the sampling frame more efficient by removing blocks of numbers that have a high chance of not being connected or are assigned to large businesses [3, 27]. These RDD methods do not include mobile or VoIP telephone numbers. Since the turn of this century, there has been a trend of households moving away from traditional landline telephones with the emergence of mobile-only households [11, 13, 15, 28]. This is due to increasing portability, flexibility, affordability and broadening internet capability of mobile telephones including smartphones and other telecommunications, such as VoIP [11, 15, 26, 29–32].

As a result of the increasing use of mobile telephones, conducting telephone surveys has become increasingly problematic in Australia and other countries [15, 33]. This is because of the difficulty in obtaining a representative sampling frame of mobile telephones numbers since are they are rarely listed (7.3 % of mobile telephone owners in South Australia are listed [26]). Unlike the structure of landline telephone numbers, the Australian mobile numbers do not provide details of geographical location and the common methods used to generate a RDD sample of landline telephone numbers geographically are not applicable to mobile telephone numbers [34, 35]. In 2011–12, approximately 20 % of households in Australia were mobile-only [14, 29], 34 % of USA households in 2012 were mobile-only [30] with countries in Europe reporting 50–70 % [32]. More notably, studies have found that mobile-only households are demographically different to traditional landline households: they are generally younger people, unrelated, never married, and socioeconomically disadvantaged [26, 30]. These issues suggest that by excluding mobile-only households biased estimates may be produced from chronic disease and behavioural risk factor surveillance systems.

This study presents the most up-to-date estimates available on the current status and possible sample biases of the current telephone survey methodology in South Australia, a state of Australia. Data from an annual representative face-to-face (non-telephone) population survey that included questions about telephone ownership were used to assess the population that were non-contactable by current telephone sampling methods. This included both household landline and mobile telephone ownership and listings in the telephone directory. This study will 1) explore trends of landline and mobile telephone ownership between 2006 and 2013; 2) describe the socio-demographic characteristics of respondents living in mobile-only households between 2010 and 2013; and 3) investigate the coverage bias of the two telephone samples (directory-listed numbers (EWP), consisting mainly of landline telephone numbers and a small proportion of mobile and VoIP telephone numbers; and a RDD sample of landline telephone numbers which excludes mobile-only households) by examining the prevalence estimates of health status and health risk behaviours between 2010 and 2013. This is one of the few studies to assess the potential bias of health estimates due to coverage bias from telephone sampling frames in terms of health indicators and socio-demographics, using a unique data source with telecommunication information on people who would be excluded from the hypothetical telephone samples [26, 30]. This study uses relatively current data, which is important since telecommunications technologies have rapidly changed and evolved over the last 10 years, with increased uptake and saturation of mobile telephones and associated changes in the way people communicate [36]. Methodological studies therefore need to continually assess sample coverage and potential bias in health-related estimates [26].

## Methods

### Survey design and sample selection

The Health Omnibus Survey (HOS) [37, 38] is a multi-stage, systematic, clustered area sample of South Australian households where face-to-face interviews are conducted annually. The HOS sample includes households randomly selected from Australian Bureau of Statistics (ABS) collector districts (CDs) (2006 to 2012) and Statistical Areas Level 1 (SA1) (2013), from the metropolitan Adelaide area and country towns with a population of 1,000 people or more. Within each CD or SA1, a random starting point was selected and from this point 10 households were selected in a given direction with a fixed skip interval. Hotels, motels, hospitals, hostels and other institutions were excluded from the sample. An approach letter and a brochure introducing the survey were sent to the selected household and the person aged 15 years or over, with the last birthday, was chosen for interview. The interviews were conducted in people’s homes by trained interviewers. Up to six call back visits were made to chosen households to interview the selected person. There was no replacement for non-respondents and no incentive of any kind was offered. Approximately 3000 people participate annually, achieving a median response rate of 59.3 % (range: 52 to 60 %). The data are weighted by five year age groups, sex, and area (metropolitan Adelaide and rural/remote South Australia) to the most recent Census or Estimated Residential Population for South Australia and probability of selection within the household size to provide population estimates.

### Household telecommunications ownership

Questions regarding telecommunications services in the household, specifically, landline telephone and mobile connections, were included in the 2006 to 2013 HOS. Mobile-only households were defined if the respondent had a mobile telephone with no working landline connection to the household. Landline connections did not include using VoIP connection or Skype for telephone calls. In addition, questions were asked regarding landlines and mobile telephones currently listed in the Australian White Pages. From these questions, household landline and mobile telecommunication status were determined by classifying the respondents as living in mobile-only households; landline-only households; landline and mobile telephone households; or having no landline or mobile in the household.

### Socio-demographics

Demographic variables included age, sex, area of residence, country of birth, household size, household structure, educational attainment, marital status, gross annual household income, employment status, dwelling ownership or renting status (2013 only) and area-level socio-economic status. The Socio-Economic Indexes for Areas (SEIFA) Index of Relative Socio-Economic Disadvantage (IRSD) is a composite score of relative disadvantage developed by the ABS [39] for particular geographical areas, such as postcodes. It is based on selected 2011 Census socio-demographic variables. The SEIFA IRSD scores were grouped into quintiles for analysis where the highest quintile comprised postcodes with the highest SEIFA IRSD scores (most advantaged areas).

### Comorbid conditions and health behaviours

Chronic conditions included self-reported medically confirmed diabetes (2010, 2011 and 2013 only), current asthma (2010 and 2011 only), arthritis and a current mental health condition. Self-reported health risk factor data included smoking status and obesity as determined by body mass index (BMI) which was derived from self-reported weight and height and recoded into four categories (underweight, normal weight, overweight and obese) [40].

### Statistical analyses

Data analysis was conducted using Stata Version 12.0. All estimates and analyses were conducted using *svy* commands in Stata to incorporate the sampling design. Univariable analyses using chi-square tests compared the proportion of mobile-only households across socio-demographic variables for 2010, 2011, 2012 and 2013. Households that had no telecommunications, refused or where the status could not be determined were excluded from the analyses (*n* = 39). The univariable analyses were limited to data from 2010, since data has been previously published for earlier years [26]. Additional univariable analyses using chi-square tests were undertaken to describe the proportion of households with a landline telephone connected; the proportion of households with mobile telephones; and the proportion of households with a directory-listed telephone number (EWP). These results can be found in Additional file 1.

To explore the possibility of coverage bias of telephone surveys, two hypothetical telephone sampling frames (subsamples) were created from HOS: 1) RDD landline, that is, households that had a landline connection (mobile-only households excluded); and 2) directory-listed numbers, that is, households with either a landline or mobile telephone number listed in the White Pages. Prevalence estimates of health conditions and behavioural risk factors were presented for the overall population, and the two hypothetical telephone samples. The hypothetical telephone samples were subsamples of the total sample (landline RDD sample is 72–78 % of the total sample and directory-listed landline sample is 50–60 % of the total sample) which means that these subsamples would have a different demographic profile to each other and the overall sample. Therefore the data for the hypothetical telephone samples were re-weighted to produce health estimates that are reflective of the South Australian population. Re-weighting is calculated by incorporating the original relative sample weights, and by age, sex and area of residence to the most recent Census or Estimated Residential Population for South Australia.

To determine the amount of bias of the prevalence estimates derived from the two hypothetical sampling frames, the relative coverage bias (RCB) was calculated by the following formula: $\frac{N_{nc}}{N}\cdot \frac{\left({p}_c - {p}_{nc}\right)}{P}$ [41]. This formula incorporates the proportion of the population that is not included in the hypothetical samples (*N*_*nc*_/*N*), that is, 1) mobile-only households, and 2) households that do not have either a mobile or landline telephone number listed in the telephone directory (*N*_*nc*_ denotes the number in the sample that is not covered in the total sample, *N*). It also includes the differences in prevalence estimate obtained from the hypothetical samples, *p*_*c*_, and from the sample not in the hypothetical samples, *p*_*nc*_, divided by the prevalence estimate for the total population, *P*.

## Results

**Fig. 1:**
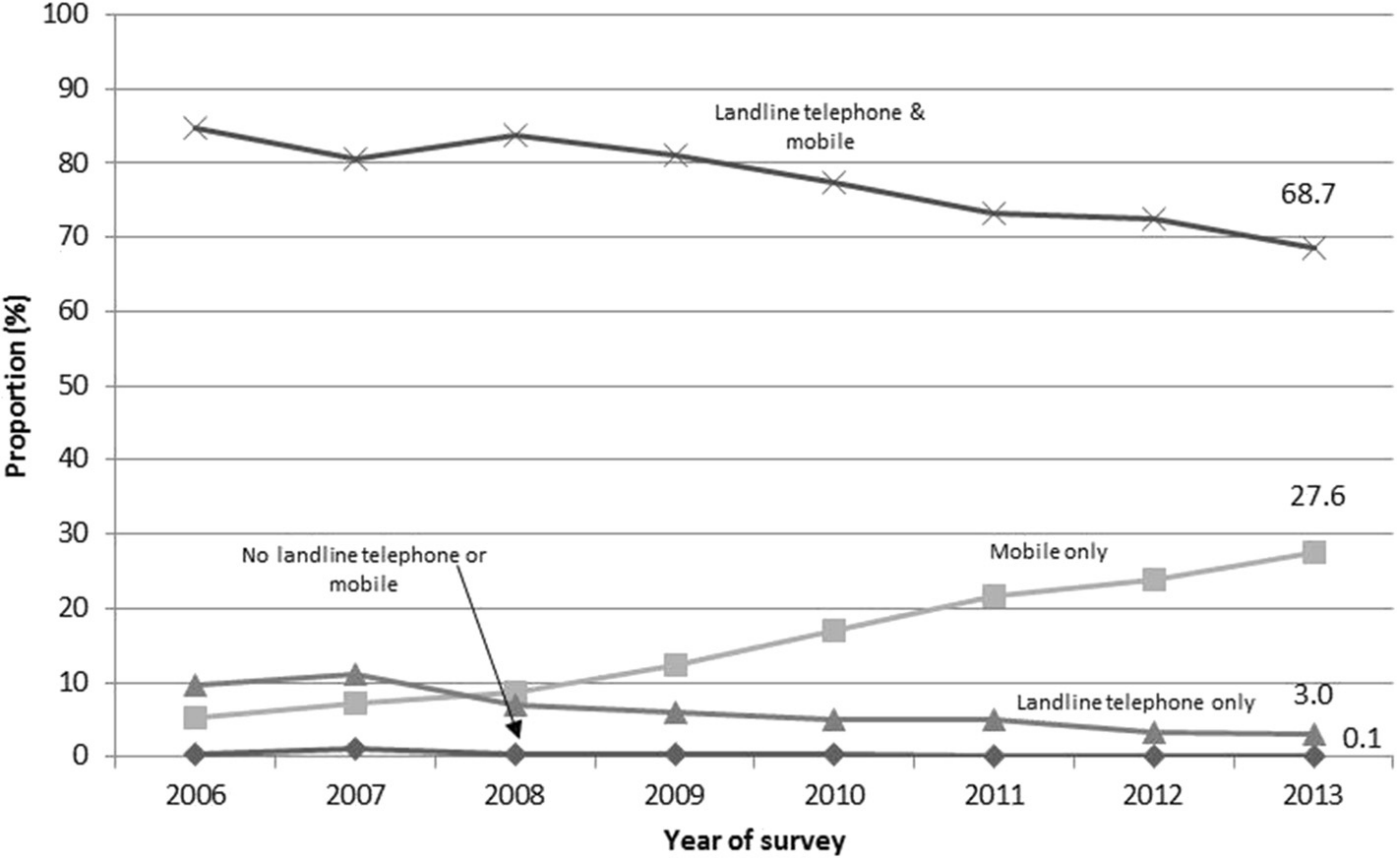
Household landline and mobile telephone status, South Australia, 2006 to 2013

Figure 1 shows the household landline and mobile telephone status from 2006 to 2013. Mobile telephone ownership was consistently around 90 % during the last eight years, rising from 89.7 % (95 % CI 88.5–90.9) in 2006 to 96.3 % (95 % CI 95.5–97.0) in 2013 (7.4 % increase). The proportion of households that are mobile-only has increased by 431 % over the eight year period from 5.2 % (95 % CI 4.4–6.0) in 2006 to 27.6 % (95 % CI 24.7–30.7) in 2013. In contrast, the proportion of landline ownership (households with landline telephone only, and households with both landline and mobile telephones) has decreased by 24.1 % from 94.4 % (95 % CI 93.2–95.) in 2006, 87.3 % (95 % CI 85.7–88.8) in 2009 to 71.7 % (95 % CI 68.6–74.6) in 2013. Descriptive statistics for the participants for 2010 to 2013 are provided in Additional file 1: Table S1.

Table 1 shows the proportion of respondents living in mobile-only households by socio-demographic variables across the four years. Generally, respondents living in mobile-only household were more likely to be male, younger, of Aboriginal or Torres Strait Islander descent, born in Asia or countries other than Australia, UK, Ireland or Europe, never married, or separated or divorced, unemployed, fulltime employed, or home duties, renting privately or from the government, and to reside in the most disadvantaged areas. Largest percentage increases over the four years occurred amongst females (86.6 %), people in the older age groups (86.2–159.4 %), people living in rural areas of South Australia (80.4 %), people born in the United Kingdom or Ireland (118.3 %), people living in single parent households or shared-care parenting households (77.7 %), or couples with no children (72.6 %), widowed (131.4 %), married or in a defacto relationship (75.2 %), people with at least secondary schooling (81.0 %), people living in households on low income levels (89.4 %) or very high income levels (97.7 %), and people who are retired (134.4 %) or who are currently students (98.6 %).

**Table 1:** Proportion of respondents living in mobile only households by socio-demographic variables, 15 years and over

		2010			2011			2012			2013	
	n	% (95 % CI)	p value	n	% (95 % CI)	p value	n	% (95 % CI)	p value	n	% (95 % CI)	p value
Sex												
Male	300	20.1 (17.5–22.9)	<0.001	339	22.8 (20.4–25.4)	0.158	391	26.2 (23.4–29.2)	0.005	409	28.8 (24.6–33.3)	0.288
Female	221	14.2 (12.3–16.3)		318	20.6 (18.1–23.3)		338	21.6 (19.5–23.9)		394	26.5 (23.6–29.6)	
Age (years)												
15 to 24	120	23.7 (18.9–29.3)	<0.001	145	28.7 (23.2–34.8)	<0.001	168	34.4 (28.4–41.0)	<0.001	171	36.9 (30.5–43.8)	<0.001
25 to 34	189	39.8 (34.7–45.1)		226	47.9 (42.4–53.4)		225	47.6 (41.8–53.6)		254	56.6 (49.2–63.7)	
35 to 44	96	18.8 (15.0–23.2)		149	29.1 (24.8–33.9)		146	28.9 (24.9–33.3)		168	35.0 (29.4–41.1)	
45 to 54	66	12.5 (9.7–15.9)		69	13.3 (10.2–17.0)		95	18.0 (14.5–22.2)		110	22.1 (17.5–27.6)	
55 to 64	31	7.0 (4.8–9.9)		36	8.0 (5.3–12.0)		69	14.9 (11.6–19.0)		61	13.7 (11.1–16.7)	
65 to 74	10	3.2 (1.9–5.3)		25	7.6 (5.4–10.6)		20	5.5 (3.8–7.7)		29	8.3 (6.1–11.2)	
75+	7	2.9 (1.5–5.5)		6	2.6 (1.3–5.1)		7	2.8 (1.4–5.4)		10	4.3 (2.5–7.2)	
Area of residence												
Metropolitan	369	16.4 (14.7–18.3)	0.315	461	20.6 (18.7–22.7)	0.122	503	22.2 (20.3–24.3)	0.005	549	25.4 (22.4–28.6)	0.016
Regional	151	18.9 (14.7–23.9)		196	24.6 (20.0–29.9)		226	28.5 (24.6–32.7)		254	34.1 (27.7–41.0)	
Number of people in household												
1	95	23.6 (20.6–26.8)	0.001	125	29.7 (26.4–33.3)	0.001	117	28.6 (23.4–34.4)	0.064	135	36.0 (31.2–41.0)	<0.001
2	267	16.7 (14.5–19.2)		352	22.5 (20.4–24.8)		397	24.9 (22.6–27.3)		433	28.6 (25.3–32.2)	
3	110	19.0 (15.2–23.3)		84	16.7 (12.7–21.7)		110	20.3 (16.3–25.1)		140	27.2 (21.6–33.6)	
4 or more	47	10.2 (6.6–15.6)		96	17.5 (12.8–23.4)		106	20.7 (15.9–26.5)		95	18.8 (14.9–23.4)	
Country of birth												
Australia	396	17.4 (15.5–19.5)	<0.001	488	22.0 (19.7–24.4)	<0.001	537	23.7 (21.6–26.0)	<0.001	595	27.9 (24.8–31.1)	0.002
UK or Ireland	25	9.3 (6.2–13.7)		39	13.7 (10.2–18.3)		61	17.9 (14.3–22.3)		60	20.3 (14.3–28.0)	
Europe	13	8.9 (5.3–14.6)		17	10.6 (6.6–16.7)		9	7.4 (3.9–13.8)		21	14.5 (10.8–19.1)	
Asia	47	29.8 (21.3–39.9)		78	32.9 (25.8–40.7)		81	41.0 (31.7–50.9)		74	35.8 (24.2–49.4)	
Other	39	19.9 (13.4–28.5)		34	26.4 (18.2–36.7)		41	31.7 (22.7–42.3)		53	42.4 (30.2–55.6)	
Aboriginal/Torres Strait Islander												
No	502	16.8 (15.0–18.7)	0.029	627	21.1 (19.2–23.1)	<0.001	686	23.1 (21.3–25.0)	<0.001	764	27.1 (24.3–30.0)	<0.001
Yes	18	35.2 (21.9–51.2)		29	53.2 (38.6–67.3)		39	51.5 (36.1–66.5)		36	53.9 (38.4–68.8)	
Household structure												
Couple family children	135	12.6 (10.3–15.5)	<0.001	209	18.1 (15.4–21.2)	<0.001	232	21.2 (18.1–24.6)	<0.001	227	21.6 (18.0–25.6)	<0.001
One parent family, other	79	22.9 (18.2–28.3)		92	31.2 (25.4–37.7)		88	29.7 (24.7–35.1)		136	40.7 (35.0–46.7)	
Lone adult person	77	21.5 (18.3–25.1)		99	26.8 (23.3–30.7)		95	26.5 (21.7–31.9)		99	31.2 (26.6–36.3)	
Couple with no children	89	11.3 (9.0–14.1)		126	15.7 (13.2–18.6)		145	16.9 (14.3–19.9)		138	19.5 (16.7–22.6)	
Other	139	28.8 (23.7–34.5)		131	31.6 (26.1–37.6)		169	37.7 (31.5–44.4)		203	40.7 (34.5–47.2)	
Marital status												
Married/defacto	253	13.3 (11.5–15.3)	<0.001	335	17.9 (15.8–20.2)	<0.001	390	20.5 (18.2–22.9)	<0.001	417	23.3 (20.1–26.8)	<0.001
Separated/Divorced	46	21.4 (17.2–26.3)		75	30.5 (25.5–36.0)		88	33.6 (28.4–39.2)		91	34.4 (30.0–39.1)	
Widowed	9	5.1 (2.9–8.7)		12	7.6 (4.8–12.0)		14	8.1 (5.3–12.1)		16	11.7 (8.3–16.4)	
Never married	207	28.1 (24.1–32.5)		233	31.0 (26.8–35.7)		237	33.4 (28.9–38.3)		279	38.9 (33.9–44.1)	
Educational attainment												
Secondary schooling	212	15.3 (13.0–17.9)	0.128	268	21.5 (18.4–25.0)	0.819	292	23.3 (20.3–26.7)	0.702	329	27.7 (23.3–32.6)	0.745
Trade, certificate, diploma	192	18.7 (16.2–21.5)		246	21.2 (18.7–23.8)		272	24.3 (21.6–27.3)		293	27.6 (24.2–31.2)	
Bachelor degree or higher	115	18.3 (15.3–21.8)		141	22.9 (19.4–26.8)		165	24.2 (20.7–28.1)		181	27.7 (23.4–32.5)	
Gross annual household income												
Up to $20,000	45	16.0 (11.4–22.0)	0.073	47	17.3 (13.3–22.2)	0.042	58	24.4 (19.7–29.7)	0.25	53	30.3 (23.9–37.4)	0.005
$20,001 – $40,000	71	17.3 (13.7–21.5)		80	20.0 (16.0–24.7)		87	25.2 (20.7–30.4)		80	21.6 (16.6–27.6)	
$40,001 – $80,000	114	18.8 (15.4–22.7)		157	26.5 (22.8–30.7)		161	27.6 (23.8–31.8)		189	33.6 (28.9–38.5)	
$80,001 – $120,000	106	21.0 (17.2–25.5)		117	24.3 (20.1–29.1)		99	24.0 (19.6–29.0)		126	28.8 (23.9–34.3)	
$120,001 or more	57	13.1 (9.8–17.1)		86	19.1 (15.1–23.8)		115	21.7 (17.8–26.1)		151	25.9 (21.6–30.7)	
Not stated	128	15.8 (12.9–19.1)		169	20.3 (16.2–25.2)		210	22.1 (18.7–25.8)		203	26.1 (22.2–30.5)	
Employment status												
Fulltime employed	248	21.9 (19.1–25.1)	<0.001	315	26.9 (24.0–30.1)	<0.001	309	27.7 (24.7–31.0)	<0.001	371	36.2 (30.9–41.9)	<0.001
Parttime employed	108	18.4 (14.6–22.9)		128	22.0 (18.2–26.2)		135	24.5 (20.5–28.9)		157	26.5 (22.2–31.3)	
Home Duties	46	22.8 (17.5–29.3)		55	32.7 (25.4–40.9)		58	27.6 (21.8–34.3)		55	34.4 (27.1–42.6)	
Unemployed	23	35.8 (23.7–50.2)		32	35.2 (24.1–48.1)		51	57.7 (45.4–69.2)		40	42.0 (28.4–56.8)	
Retired	20	3.2 (2.1–4.9)		35	5.7 (4.1–7.9)		36	5.9 (4.4–7.7)		44	7.5 (5.5–10.2)	
Student	43	14.7 (10.2–20.7)		51	21.1 (14.3–29.9)		98	29.7 (23.0–37.3)		81	29.2 (23.3–36.0)	
Other/not working due to health	32	20.0 (13.0–29.5)		38	23.4 (18.1–29.7)		41	28.8 (21.6–37.2)		53	32.6 (25.1–41.2)	
SEIFA IRSD quintile												
Lowest (most disadvantaged)	160	22.3 (18.1–27.1)	0.003	197	27.3 (23.4–31.5)	<0.001	227	30.3 (26.6–34.3)	<0.001	229	34.6 (28.1–41.7)	0.004
Low	86	17.8 (14.9–21.1)		179	29.3 (24.5–34.6)		137	26.8 (22.2–32.0)		186	31.0 (25.8–36.8)	
Middle	106	17.0 (13.5–21.3)		110	20.6 (16.8–25.0)		139	25.2 (21.2–29.6)		142	28.0 (21.8–35.1)	
High	84	15.1 (11.8–19.1)		81	15.7 (12.7–19.3)		110	19.8 (16.2–24.0)		120	22.2 (16.8–28.7)	
Highest (least disadvantaged)	84	12.6 (9.8–16.1)		91	13.9 (11.2–17.1)		116	16.9 (13.8–20.4)		125	21.0 (17.5–25.0)	
Dwelling status												
Owned or being purchased										396	18.3 (16.1–20.7)	<0.001
Rent from state government (public housing)										61	43.5 (34.5–52.9)	
Rent privately										330	58.6 (52.8–64.3)	
Other										9	33.4 (15.7–57.6)	
Overall	520	17.1 (15.3–19.0)		657	21.7 (19.7–23.8)		729	23.9 (22.0–25.9)		803	27.6 (24.7–30.7)	

Additional file 1: Tables S2 and S3 show the proportion of respondents living in a household with a landline connection and the proportion of respondents living a household with at least one mobile telephone by socio-demographic variables for 2010 to 2013. The proportion of respondents living in households with directory-listed mobile or landline telephone (EWP) has been steadily decreasing from 73.8 % (95 % CI 72.2–75.4) in 2006, to 60.4 % (95 % CI 58.1–62.7) in 2010 and 49.6 % (95 % CI 46.2–53.0) in 2013. This proportion by socio-demographic characteristics for 2010 to 2013 is listed in Additional file 1: Table S4. In 2013, 4.6 % (95 % CI 3.8–5.5) of mobile numbers were listed in the telephone directory compared to 62.7 % (95 % CI 59.2–66.1) of landlines.

The prevalence estimates of various health conditions and behavioural risk factors for all households, for people who live in households with a landline connection (hypothetical landline RDD sample) and for people who live in a household with a directory-listed landline or mobile telephone number (hypothetical directory-listed sample) are shown in Table 2. The RCB for the prevalence estimates derived from the two hypothetical samples are also in Table 2. There were small absolute differences in the prevalence estimates for current asthma, arthritis and obesity between the hypothetical telephone samples and the overall sample. The prevalence estimates for diabetes by the two hypothetical samples did not differ in 2010 and 2011, however, the prevalence estimate was slightly underestimated (RCB value of −0.077) in 2013 for the directory-listed sample. Even though the prevalence estimates for arthritis were similar for both hypothetical samples, the prevalence estimate for arthritis in 2010 was underestimated for the directory-listed sample (RCB value of −0.083) compared to the overall sample (prevalence of 20.7 vs. 21.4 %). The prevalence of having a mental health condition showed mixed results for both hypothetical samples and over time: the prevalence of having a mental health condition was underestimated for both samples with estimates from the directory-listed sample having larger RCB (ranging from −0.102 to −0.242) with the exception of 2011, which had the opposite result of overestimating mental health conditions (RCB value of 0.056). Current smoking prevalence was lower for both hypothetical telephone samples with absolute differences ranging from 2.9 to 3.4 percentage points for RDD landline samples and 3.3 to 5.3 percentage points for directory-listed samples, and associated large RCB values: −0.136 to −0.191 for RDD landline samples and −0.129 to −0.313 for directory-listed samples.

**Table 2:** Prevalence of health conditions and risk factors for all households, and for landline Random Digit Dialling (RDD) and Directory-listed (EWP) telephone samples, 15 years and over

			2010			2011			2012			2013	
		n	% (95 % CI)	RCB	n	% (95 % CI)	RCB	n	% (95 % CI)	RCB	n	% (95 % CI)	RCB
	Health conditions												
Diabetes	All households	229	7.5 (6.6–8.5)		246	8.1 (7.1–9.2)					247	8.5 (7.6–9.5)	
	Landline (RDD) sample	181	7.2 (6.2–8.3)	–0.009	195	8.3 (7.1–9.6)	0.003				173	8.3 (7.4–9.4)	−0.033
	Directory-listed sample	133	7.1 (6.0–8.5)	−0.021	148	8.6 (7.1–10.3)	0.030				117	7.9 (6.7–9.4)	−0.077
Current asthma	All households	416	13.6 (12.2–15.3)		384	12.7 (11.4–14.0)							
	Landline (RDD) sample	335	13.3 (11.7–15.2)	−0.015	297	12.6 (11.1–14.2)	−0.002						
	Directory-listed sample	246	13.1 (11.3–15.2)	−0.061	220	12.8 (10.9–15.0)	−0.014						
Arthritis	All households	653	21.4 (19.9–23.1)		727	24.0 (22.3–25.7)		656	21.5 (19.7–23.3)		640	22.0 (20.3–23.8)	
	Landline (RDD) sample	529	21.1 (19.5–22.8)	−0.025	554	23.5 (21.6–25.4)	−0.031	495	21.2 (19.3–23.2)	0.009	464	22.3 (20.4–24.4)	0.019
	Directory-listed sample	387	20.7 (18.9–22.6)	−0.083	407	23.7 (21.4–26.1)	−0.018	346	20.7 (18.5–23.2)	−0.046	324	22.0 (19.7–24.4)	−0.016
Mental health condition	All households	330	10.8 (9.6–12.2)		359	11.8 (10.7–13.1)		297	9.7 (8.7–10.8)		384	13.2 (11.8–14.7)	
	Landline (RDD) sample	250	10.0 (8.6–11.5)	−0.100	275	11.6 (10.3–13.1)	−0.046	222	9.5 (8.2–10.9)	−0.025	256	12.3 (10.7–14.1)	−0.091
	Directory-listed sample	167	8.9 (7.4–10.7)	−0.242	221	12.9 (11.0–15.0)	0.056	150	9.0 (7.6–10.6)	−0.102	172	11.6 (9.4–14.3)	−0.141
	Health - related risk factors												
Current smoker	All households	614	20.2 (18.3–22.1)		529	17.4 (15.8–19.2)		501	16.4 (14.8–18.2)		552	19.0 (16.6–21.6)	
	Landline (RDD) sample	429	17.1 (15.2–19.2)	−0.136	343	14.5 (12.8–16.5)	−0.191	304	13.0 (11.4–14.9)	−0.174	330	15.9 (13.6–18.6)	−0.138
	Directory-listed sample	316	16.9 (14.7–19.3)	−0.129	223	13.0 (10.9–15.4)	−0.313	210	12.6 (10.8–14.7)	−0.181	202	13.7 (11.6–16.1)	−0.244
Obese	All households	611	22.1 (20.4–24.0)		612	22.7 (20.9–24.6)		621	23.4 (21.5–25.4)		603	23.1 (20.7–25.8)	
	Landline (RDD) sample	511	22.6 (20.7–24.6)	0.036	491	23.3 (21.2–25.5)	0.013	474	23.5 (21.4–25.8)	−0.020	454	24.3 (21.8–27.0)	0.078
	Directory-listed sample	379	22.5 (20.3–25.0)	−0.002	359	23.8 (21.2–26.6)	0.059	329	22.7 (20.1–25.5)	−0.050	329	24.6 (21.6–27.9)	0.079

Note: *RCB* relative coverage bias, *Landline (RDD) sample* households that had a landline connection (mobile-only households excluded); *Directory-listed sample* households with either a landline or mobile telephone number listed in the White Pages

## Discussion

This paper presents estimates and trends of telephone coverage in Australia from 2006 to 2013. Continual assessment of methodological issues around conducting population health telephone surveys is essential due to the rapid technological changes in telecommunications and the different ‘user culture’ associated in use of these new and old telecommunication technologies. Even though telephone (landline and mobile) coverage in South Australia is very high (97 %), nearly a third of households are mobile-only (27.8 %) and only half of the households (49.0 %) have either a mobile or landline number listed in the White Pages telephone directory. Our results show that mobile-only respondents are different across a range of socio-demographic indicators, which is similar to international studies [13, 15, 30]. Using hypothetical sampling frames (RDD landline and EWP directory listing) that were weighted to the age and sex structure of the South Australian population produced contradictory results for health prevalence estimates when compared to all households in the face-to-face survey. Prevalence estimates of diabetes, current asthma, arthritis and obesity had very minor differences and biases, but the prevalence estimates for mental health condition and current smoking indicates biases using either RDD landline or EWP directory listing sampling frame. Even though our results show that mobile-only respondents are demographically different across a range of socio-demographic indicators, appropriately weighted data can produce reliable prevalence estimates for some health indicators, but not for others. These findings suggest landline-based sampling frames used in Australia are potentially biased for some health indicators, such as current smokers and having a mental health condition, particularly where conditions or risk factors are higher amongst those living in mobile-only households. Researchers using either RDD or directory-listing landline sampling frames need to be aware of their limitations and know of the potential biased estimates because of the groups that are excluded from the sampling frames.

This study is important because it quantifies the potential biases from the various landline-based telephone sampling frames used in Australia and the groups that are potentially excluded. Even though the data are limited to South Australia, the conclusions may be generalizable to the Australian population. This study is unique since the same questions have been asked annually for eight years and, using the face-to-face methodology in which all types of households are included (mobile-only, landline-only or both), it had the ability to examine, over time, the prevalence estimates of various health indicators by telephone status. Very few studies like this are known to exist nationally [14] and internationally [15, 30] and even fewer examine the assessment on health indicators [30].

The trends and demographic differences found in this study are similar to national and international studies [11, 14, 15, 30, 42, 43] and support findings from our previous research [26]. Our estimate of mobile-only households in 2012 (23.9 %) was higher than the estimate reported by the Australian Communication and Media Authority (19 %) [14]; the proportion of households with a landline telephone in 2010 was 82.5 % which was slightly higher than the 80.3 % estimate from the 2010–11 Australian Health Survey (AHS); and our estimate of 68.7 % of landline telephone numbers listed in the telephone directory was slightly lower than the 70.1 % from the AHS 2010–11 survey [44]. Between 2006 and 2008 the trend of mobile-only households remained low, however since 2009, the trend has steadily increased, following international patterns [30]. Similarly for landline ownership, up to 2011 the proportion was over 80 %, however, this has steadily decreased to 71.9 % in 2013. These changes are mainly due to the increasing popularity of greater flexibility and affordability offered by mobile technology. People are using landlines less frequently because they are able to have a single device with multiple communication and media services, which is less expensive than having a landline connection [13].

In our previous study [26], nearly 10 % of the population in 2008 lived in mobile-only households, and we showed that with appropriate weighting, the sampling methodology used for telephone surveys produced reliable health estimates with the exception of smoking prevalence in South Australia being underestimated. In contrast, with more recent data and up-to-date analyses, this study has estimated that close to 30 % of the Australian population now live in mobile-only households and these analyses have demonstrated the impact of the vast changes in the telecommunication over the eight year study period on the coverage of the sampling frames. Excluding a distinct subpopulation from the landline sampling frames, namely mobile-only households, resulted in under- or over-estimation in some health estimates, although with appropriate weighting most health estimates (except smoking and mental health) were very similar to the overall population. Even though the results in the health estimates (absolute differences and RCB values) between the overall population and the two hypothetical landline sample groups showed no clear pattern over time, the results do highlight that for specific health indicators, such as current smokers and mental health, the direction of the bias was consistently under-estimated for both RDD and directory-listed landline hypothetical samples. The other conditions (diabetes, current asthma, arthritis and obesity) had little absolute differences in health estimates and an inconsistent pattern, but relatively low, RCB values over time, which may suggest that the differences could be due to the random nature of the sample or other sampling errors. Our findings for current smokers, asthma and obesity are similar to other USA studies [30] using similar methodology, and are consistent with studies using dual-frame telephone surveys for mental health [45], current smoking [30, 46, 47], asthma [47], and obesity [30]. This suggests that perhaps an alternative sampling, surveying or statistical methodological approach may need to be considered to include groups of the population to remove the coverage biases in landline-based sampling frames.

Many studies have explored various methods to include the mobile-only group into chronic disease and risk factor surveillance systems [12, 48]. The favoured method is an over-lapping dual-frame design which involves two independent samples: a sample of mobile telephones and a landline-based sample [34, 35, 46, 49]. These studies showed an improvement in the representativeness, in particular for men, the younger and middle age groups, and people who were never married. However, obtaining a sample of mobile telephone numbers does have drawbacks, including low response rates and two to four times the costs of landline-based samples [34]. More importantly, the mobile sample that is currently available and used in Australia is of randomly generated mobile telephone numbers with no geographical marker. From a South Australian perspective, only 8 % of all mobile telephone numbers in Australia were estimated to be owned by South Australians [34, 35, 46, 49], which is almost the same proportion of the state’s population (7.4 %). This means a much larger initial sample is required for screening, and with the additional problem of low response rate, the feasibility of including mobile numbers using these methods in a chronic disease and behavioural risk factor surveillance system in South Australia would be costly. Even though 98 % of South Australians have a mobile telephone and it is perceived that people can be reached anytime, it does not mean that they are willing or able to use it to complete a survey. Receiving mobile telephone calls can happen at unpredictable moments when it is not suitable for the owner to respond, such as driving (safety issue), travelling overseas (which can incur a large cost to the researcher or participant), or during a meeting or in a restaurant (privacy issue); all have an impact on response rates [43].

Mixed-mode methods have also been suggested as a way to complement the traditional landline telephone survey by combining face-to-face, mail, and internet surveys [50]. These alternative modes introduce other methodological issues and the design of each mode need to be taken into consideration. The questionnaire design for CATI surveys, for example, complicated skips patterns or data range checks, needs to be careful considered in other modes such as mail survey [51]. Face-to-face, mail and internet survey can have the option of longer worded questions, explanations, and visual or prompt cards which is not recommended or possible with CATI surveys. Therefore, the wording of the questions in telephone surveys needs to be clear, concise and short [52]. Operational differences can have an impact on how the questions are answered. Telephone surveys are mainly interviewer administered whereas mail or internet surveys are self-administered which can lead to different responses [50, 51]. In telephone surveys, the interviewer has control over who is the selected respondent within the household whereby in the mail or internet surveys any member of the household determines who is the selected [12]. The level of privacy can vary by survey modes which is high with mail or internet surveys compared to moderate level of privacy with telephone (others listening in, or answering sensitive questions) [53]. Mail surveys require a longer data collection period compared to the allocated time period for telephone surveys. In an attempt to include respondents from mobile-only households, a study examined the possibly of using two modes, telephone and mail, with a single database that consisted of residential addresses. However, they found that the groups that were under-represented in telephone surveys were also under-represented in the mail surveys [48]. Another consideration for surveillance systems that used the telephone to collect data, is the challenge of how to incorporate alternative modes but still maintain the timeliness, flexibility, low non-response and low cost of the system [12]. Other methodological studies have used statistical approaches such as alternative weighting strategies, such as raked weights, which incorporate a wider range of socio-demographic variables, can improve the health estimates and are more in line with face-to-face surveys [54–56].

The study design used in this research is robust due to the large representative state-wide samples used and is unique in that the data were collected over eight years using the same or similar questions, and by one organisation, thus minimising interviewer biases. These data are also very recent and it is one of the few face-to-face studies conducted in Australia and worldwide that included questions on landline and mobile telephone status that also had questions on health status and behavioural risk factors [30] so the biases in health estimates can be assessed. However the results could be biased due to the moderately acceptable response rates (median = 59.3 %) which is following the trends observed interstate and overseas. This study only analysed a few health-related variables and additional questions such as health service usage, quality-of-life or alcohol consumption would have provided a more comprehensive description of telephone sampling biases.

## Conclusion

Telephone surveys have become a standard and accepted method of collecting health information in Australia and are widely used to monitor chronic disease and behavioural risk factors. Such surveillance systems provide evidence to inform interventions and service planning with the aim of reducing the impact of chronic diseases and their associated costs to the health system. Analyses like those presented here are important to demonstrate that the health estimates obtained are not biased due to sampling methodology. This study has shown that the proportion of mobile-only households is increasing and this does not appear to have reached a plateau. This corresponds with the decrease in landline telephone coverage. Even with appropriately weighted data, using landline-based sampling frames in Australia are potentially biased for some health indicators. This implies that the landline sampling frames that are currently used in most Australian chronic disease and risk factor surveillance systems (RDD landline or directory-listed telephone numbers) are not sufficient on their own because of the exclusion of the mobile-only households. Other methodologies need to be considered for small states like South Australia that are timely, cost-effective and efficient.

### Availability of data and materials

The Health Omnibus Survey (HOS) is a user-pay survey in which various organisations pay for their questions to be included in the surveys. Because of this, the authors of this study do not own all of the HOS data and permission had to be sought from each owner, therefore data are not publicly available.

### Ethical statement

Ethical approvals were obtained from the Research Ethics Committees of The University of Adelaide and the South Australian Department of Health. Participation in the study is voluntary. Verbal informed consent was obtained from participants at the start of the interview. Prior to contact by the interviewers, a primary approach letter was sent to the household informing the household of the purpose of the survey including a pamphlet listing the organisations involved in the survey, confidentiality and privacy assurance, that participation is voluntary, and a contact number for queries. Upon initial contact, the interviewers repeat the purpose of the survey as well as the expected length of time to complete the interview.

### Consent for publication

Not applicable.

## Additional file

Additional file 1: Table S1. Sample socio-demographic profile by survey year, 15 years and over. **Table S2.** Proportion of respondents living in households with a landline connection (RDD) by socio-demographic variables, 15 years and over. **Table S3.** Proportion of respondents living in households with at least one mobile telephone by socio-demographic variables, 15 years and over. **Table S4.** Proportion of respondents living in households with a directory-listed telephone number (EWP) by socio-demographic variables, 15 years and over. (DOCX 107 kb)

## Abbreviations

ABS: Australian Bureau of Statistics
BMI: body mass index
CATI: computer assisted telephone interviewing
CD: collector districts
EWP: electronic white pages
HOS: Health Omnibus Survey
IRSD: index of relative social disadvantage
RCB: relative coverage bias
RDD: random digit dialling
SA1: Statistical Areas Level 1
SEIFA: Socio-Economic Indexes for Areas
SPSS: Statistical Package for Social Sciences
VoIP: Voice over Internet Protocol
