# Supplementary material for: Bias of health estimates obtained from chronic disease and risk factor surveillance systems using telephone population surveys in Australia: results from a representative face-to-face survey in Australia from 2010 to 2013
Source: BMC Med Res Methodol. 2016 Apr 18;16:44. doi: 10.1186/s12874-016-0145-z (PMC4836184; doi:10.1186/s12874-016-0145-z)
Supplement: Additional file 1: Table S1. — Sample socio-demographic profile by survey year, 15 years and over. Table S2. Proportion of respondents living in households with a landline connection (RDD) by socio-demographic variables, 15 years and over. Table S3. Proportion of respondents living in households with at least one mobile telephone by socio-demographic variables, 15 years and over. Table S4. Proportion of respondents living in households with a directory-listed telephone number (EWP) by socio-demographic variables, 15 years and over. (DOCX 107 kb) [file 12874_2016_145_MOESM1_ESM.docx]

Table S1: Sample socio-demographic profile by survey year, 15 years and over

|  |  | **2010** |  | **2011** |  | **2012** |  | **2013** |
| --- | --- | --- | --- | --- | --- | --- | --- | --- |
|  | **n** | **% (95% CI)** | **n** | **% (95% CI)** | **n** | **% (95% CI)** | **n** | **% (95% CI)** |
| **Sex** |  |  |  |  |  |  |  |  |
| Male | 1493 | 49.0 (46.8 - 51.2) | 1486 | 49.0 (47.2 - 50.9) | 1494 | 48.9 (46.8 - 50.9) | 1422 | 48.9 (46.5 - 51.3) |
| Female | 1553 | 51.0 (48.8 - 53.2) | 1546 | 51.0 (49.1 - 52.8) | 1561 | 51.1 (49.1 - 53.2) | 1486 | 51.1 (48.7 - 53.5) |
| **Age (years)** |  |  |  |  |  |  |  |  |
| 15 to 24 | 507 | 16.7 (14.9 - 18.4) | 505 | 16.7 (14.8 - 18.5) | 487 | 15.9 (14.1 - 17.8) | 464 | 15.9 (14.4 - 17.7) |
| 25 to 34 | 475 | 15.6 (14.0 - 17.2) | 473 | 15.6 (14.0 - 17.2) | 472 | 15.4 (13.8 - 17.1) | 449 | 15.4 (13.2 - 18.0) |
| 35 to 44 | 513 | 16.9 (15.2 - 18.5) | 511 | 16.9 (15.3 - 18.4) | 505 | 16.5 (14.8 - 18.3) | 480 | 16.5 (15.1 - 18.0) |
| 45 to 54 | 525 | 17.2 (15.7 - 18.7) | 523 | 17.2 (15.8 - 18.7) | 525 | 17.2 (15.5 - 18.9) | 499 | 17.2 (15.5 - 19.0) |
| 55 to 64 | 452 | 14.8 (13.4 - 16.3) | 450 | 14.8 (13.5 - 16.1) | 466 | 15.2 (13.7 - 16.8) | 443 | 15.2 (13.9 - 16.7) |
| 65 to 74 | 318 | 10.4 (9.2 - 11.6) | 327 | 10.8 (9.6 - 12.0) | 362 | 11.8 (10.6 - 13.1) | 346 | 11.9 (10.8 - 13.1) |
| 75+ | 255 | 8.4 (7.4 - 9.4) | 243 | 8.0 (7.1 - 9.0) | 239 | 7.8 (6.7 - 9.0) | 226 | 7.8 (6.5 - 9.2) |
| **Area of residence** |  |  |  |  |  |  |  |  |
| Metropolitan | 2245 | 73.7 (68.8 - 78.6) | 2235 | 73.7 (68.7 - 78.7) | 2235 | 73.2 (66.7 - 79.6) | 2163 | 74.4 (65.1 - 81.9) |
| Regional | 801 | 26.3 (21.4 - 31.2) | 797 | 26.3 (21.3 - 31.3) | 820 | 26.8 (20.4 - 33.3) | 745 | 25.6 (18.1 - 34.9) |
| **Country of birth** |  |  |  |  |  |  |  |  |
| Australia | 2277 | 74.7 (72.7 - 76.8) | 2221 | 73.3 (71.3 - 75.2) | 2267 | 74.2 (72.3 - 76.1) | 2138 | 73.5 (70.5 - 76.3) |
| UK or Ireland | 267 | 8.8 (7.6 - 9.9) | 285 | 9.4 (8.2 - 10.6) | 341 | 11.2 (9.9 - 12.5) | 293 | 10.1 (7.8 - 13.0) |
| Europe | 149 | 4.9 (4.1 - 5.7) | 157 | 5.2 (4.4 - 6.0) | 122 | 4.0 (3.3 - 4.7) | 147 | 5.1 (3.9 - 6.6) |
| Asia | 157 | 5.2 (4.0 - 6.3) | 239 | 7.9 (6.5 - 9.3) | 197 | 6.5 (5.1 - 7.8) | 206 | 7.1 (5.0 - 10.0) |
| Other | 196 | 6.5 (5.3 - 7.6) | 130 | 4.3 (3.4 - 5.2) | 128 | 4.2 (3.3 - 5.0) | 124 | 4.3 (3.4 - 5.3) |
| **Household structure** |  |  |  |  |  |  |  |  |
| Couple family children | 1068 | 35.1 (32.8 - 37.4) | 1152 | 38.0 (35.8 - 40.2) | 1095 | 35.9 (33.7 - 38.0) | 1049 | 36.1 (33.4 - 38.9) |
| One parent family, other | 348 | 11.4 (10.0 - 12.8) | 295 | 9.7 (8.4 - 11.0) | 298 | 9.8 (8.2 - 11.3) | 334 | 11.5 (9.7 - 13.5) |
| Lone adult person | 359 | 11.8 (10.7 - 12.9) | 368 | 12.1 (11.1 - 13.2) | 358 | 11.7 (10.7 - 12.7) | 317 | 10.9 (9.8 - 12.1) |
| Couple with no children | 788 | 25.9 (23.5 - 28.3) | 801 | 26.4 (24.6 - 28.2) | 855 | 28.0 (26.0 - 30.0) | 709 | 24.4 (21.9 - 27.1) |
| Other | 484 | 15.9 (13.7 - 18.0) | 416 | 13.7 (11.9 - 15.5) | 448 | 14.7 (13.0 - 16.4) | 499 | 17.2 (14.0 - 20.9) |
| **Marital status** |  |  |  |  |  |  |  |  |
| Married/defacto | 1906 | 62.6 (60.4 - 64.7) | 1871 | 61.7 (59.7 - 63.7) | 1905 | 62.4 (60.3 - 64.4) | 1791 | 61.6 (59.0 - 64.1) |
| Separated/Divorced | 217 | 7.1 (6.3 - 8.0) | 246 | 8.1 (7.2 - 9.0) | 261 | 8.5 (7.6 - 9.5) | 264 | 9.1 (7.9 - 10.4) |
| Widowed | 180 | 5.9 (5.2 - 6.7) | 161 | 5.3 (4.6 - 6.0) | 176 | 5.8 (5.1 - 6.5) | 133 | 4.6 (3.9 - 5.3) |
| Never married | 736 | 24.2 (22 - 26.3) | 751 | 24.8 (22.8 - 26.7) | 710 | 23.2 (21.3 - 25.2) | 718 | 24.7 (22.4 - 27.1) |
| **Educational attainment** |  |  |  |  |  |  |  |  |
| Secondary schooling | 1386 | 45.5 (43.1 - 47.9) | 1247 | 41.1 (38.7 - 43.6) | 1251 | 41.0 (38.1 - 43.8) | 1187 | 40.8 (36.5 - 45.3) |
| Trade quals, Certificate, | 1027 | 33.7 (31.7 - 35.7) | 1162 | 38.3 (36.2 - 40.5) | 1119 | 36.6 (34.5 - 38.7) | 1061 | 36.5 (32.5 - 40.7) |
| Bachelor Degree | 629 | 20.7 (18.4 - 22.9) | 617 | 20.4 (18.5 - 22.3) | 682 | 22.3 (19.9 - 24.7) | 651 | 22.4 (18.1 - 27.4) |
| **Gross annual household income** |  |  |  |  |  |  |  |  |
| Up to $20,000 | 280 | 9.2 (8.1 - 10.3) | 274 | 9.0 (7.9 - 10.2) | 239 | 7.8 (6.7 - 8.9) | 176 | 6.0 (5.0 - 7.3) |
| $20,001 - $40,000 | 410 | 13.5 (12.1 - 14.8) | 401 | 13.2 (11.8 - 14.7) | 344 | 11.3 (10 - 12.5) | 372 | 12.8 (11.1 - 14.7) |
| $40,001 - $80,000 | 607 | 19.9 (18.2 - 21.6) | 590 | 19.5 (17.8 - 21.1) | 582 | 19.1 (17.4 - 20.7) | 562 | 19.3 (17.3 - 21.5) |
| $80,001 - $120,000 | 504 | 16.5 (14.5 - 18.6) | 481 | 15.9 (13.9 - 17.8) | 412 | 13.5 (11.9 - 15.0) | 438 | 15.1 (13.5 - 16.7) |
| $120,001 or more | 438 | 14.4 (12.7 - 16.1) | 452 | 14.9 (13 - 16.8) | 530 | 17.3 (15.3 - 19.4) | 584 | 20.1 (16.8 - 23.8) |
| Not stated | 808 | 26.5 (24.3 - 28.7) | 834 | 27.5 (25.0 - 30.0) | 948 | 31.0 (28.4 - 33.6) | 776 | 26.7 (23.7 - 29.9) |
| **Employment status** |  |  |  |  |  |  |  |  |
| Full - time employed | 1131 | 37.1 (35.1 - 39.2) | 1171 | 38.6 (36.6 - 40.6) | 1115 | 36.5 (34.3 - 38.7) | 1025 | 35.3 (32.6 - 38) |
| Part - time employed | 586 | 19.2 (17.7 - 20.8) | 582 | 19.2 (17.6 - 20.7) | 553 | 18.1 (16.5 - 19.7) | 591 | 20.3 (18.3 - 22.5) |
| Home Duties | 203 | 6.7 (5.6 - 7.8) | 168 | 5.5 (4.6 - 6.5) | 211 | 6.9 (5.5 - 8.3) | 161 | 5.5 (4.4 - 6.9) |
| Unemployed | 65 | 2.1 (1.5 - 2.7) | 90 | 3.0 (2.2 - 3.8) | 89 | 2.9 (2.1 - 3.7) | 96 | 3.3 (2.4 - 4.6) |
| Retired | 608 | 20.0 (18.2 - 21.7) | 610 | 20.1 (18.5 - 21.7) | 614 | 20.1 (18.3 - 21.9) | 593 | 20.4 (18.2 - 22.7) |
| Student | 292 | 9.6 (8.1 - 11.1) | 241 | 7.9 (6.5 - 9.4) | 330 | 10.8 (9.1 - 12.5) | 275 | 9.5 (8.2 - 10.9) |
| Other/Not working due to illness | 161 | 5.3 (4.1 - 6.5) | 164 | 5.4 (4.5 - 6.4) | 144 | 4.7 (3.8 - 5.6) | 163 | 5.6 (4.6 - 6.8) |
| **SEIFA IRSD quintile** |  |  |  |  |  |  |  |  |
| Lowest (most disadvantaged) | 716 | 23.5 (16.9 - 30.1) | 721 | 23.8 (17.0 - 30.5) | 750 | 24.5 (17.7 - 31.4) | 663 | 22.8 (14.8 - 33.5) |
| Low | 485 | 15.9 (11.2 - 20.7) | 609 | 20.1 (14.9 - 25.3) | 513 | 16.8 (11.5 - 22.0) | 600 | 20.6 (14.6 - 28.4) |
| Middle | 622 | 20.4 (15.8 - 25.1) | 532 | 17.5 (13.3 - 21.8) | 551 | 18.0 (13.8 - 22.3) | 508 | 17.5 (11.3 - 26.1) |
| High | 558 | 18.3 (14.3 - 22.4) | 513 | 16.9 (13.3 - 20.5) | 555 | 18.2 (14.3 - 22.1) | 543 | 18.7 (12.4 - 27.1) |
| Highest (least disadvantaged) | 664 | 21.8 (17.7 - 25.9) | 657 | 21.7 (17.4 - 25.9) | 686 | 22.5 (18.1 - 26.8) | 595 | 20.5 (12.7 - 31.4) |
|  | 3046 | 100.0 | 3032 | 100.0 | 3055 | 100.0 | 2908 | 100.0 |

Note: weighted sample

Table S2: Proportion of respondents living in households with a landline connection (RDD) by socio-demographic variables, 15 years and over

|  |  | **2010** |  |  | **2011** |  |  | **2012** |  |  | **2013** |  |
| --- | --- | --- | --- | --- | --- | --- | --- | --- | --- | --- | --- | --- |
|  |  | **% (95% CI)** | **p value** |  | **% (95% CI)** | **p value** |  | **% (95% CI)** | **p value** |  | **% (95% CI)** | **p value** |
| **Sex** |  |  |  |  |  |  |  |  |  |  |  |  |
| Male | 1177 | 78.8 (75.7-81.7) | <0.001 | 1144 | 77.0 (74.4-79.4) | 0.167 | 1097 | 73.5 (70.5-76.3) | 0.005 | 999 | 70.3 (65.8-74.4) | 0.184 |
| Female | 1325 | 85.4 (82.9-87.5) |  | 1224 | 79.2 (76.5-81.7) |  | 1219 | 78.1 (75.7-80.2) |  | 1086 | 73.0 (69.9-75.9) |  |
| **Age (years)** |  |  |  |  |  |  |  |  |  |  |  |  |
| 15 to 24 | 381 | 75.2 (68.9-80.6) | <0.001 | 359 | 71.1 (65.0-76.6) | <0.001 | 316 | 64.8 (58.1-71.0) | <0.001 | 289 | 62.4 (55.4-68.9) | <0.001 |
| 25 to 34 | 279 | 58.8 (53.6-63.7) |  | 247 | 52.1 (46.6-57.6) |  | 246 | 52.2 (46.2-58.1) |  | 189 | 42.0 (34.9-49.5) |  |
| 35 to 44 | 416 | 81.0 (76.5-84.8) |  | 361 | 70.7 (65.9-75.0) |  | 359 | 71.1 (66.7-75.1) |  | 310 | 64.4 (58.5-70.0) |  |
| 45 to 54 | 456 | 86.8 (83.3-89.6) |  | 452 | 86.4 (82.6-89.4) |  | 427 | 81.3 (77.1-85.0) |  | 385 | 77.1 (71.5-81.8) |  |
| 55 to 64 | 417 | 92.3 (88.9-94.7) |  | 413 | 91.8 (87.9-94.5) |  | 395 | 84.8 (80.7-88.2) |  | 381 | 85.9 (82.8-88.6) |  |
| 65 to 74 | 307 | 96.7 (94.6-98.0) |  | 302 | 92.3 (89.2-94.5) |  | 342 | 94.5 (92.3-96.2) |  | 316 | 91.4 (88.4-93.6) |  |
| 75+ | 246 | 96.4 (93.4-98.0) |  | 235 | 96.9 (94.1-98.4) |  | 231 | 96.9 (94.3-98.3) |  | 215 | 95.3 (92.2-97.3) |  |
| **Area of residence** |  |  |  |  |  |  |  |  |  |  |  |  |
| Metropolitan | 1872 | 83.4 (81.5-85.1) | 0.133 | 1772 | 79.2 (77.2-81.2) | 0.107 | 1754 | 77.6 (75.5-79.5) | 0.004 | 1599 | 73.9 (70.6-77.0) | 0.015 |
| Regional | 631 | 78.7 (71.8-84.3) |  | 597 | 75.0 (69.8-79.6) |  | 562 | 70.8 (66.4-74.9) |  | 486 | 65.2 (58.4-71.4) |  |
| **Number of people in household** |  |  |  |  |  |  |  |  |  |  |  |  |
| 1 | 301 | 74.3 (70.6-77.6) | <0.001 | 293 | 69.7 (66.0-73.1) | <0.001 | 286 | 70.1 (64.0-75.6) | 0.046 | 233 | 62.0 (57.0-66.7) | <0.001 |
| 2 | 1324 | 82.9 (80.1-85.4) |  | 1207 | 77.3 (75.0-79.4) |  | 1198 | 75.1 (72.6-77.3) |  | 1075 | 71.0 (67.5-74.3) |  |
| 3 | 464 | 79.6 (75.0-83.6) |  | 418 | 83.0 (78.1-87.1) |  | 429 | 79.3 (74.3-83.6) |  | 370 | 71.9 (65.5-77.5) |  |
| 4 or more | 414 | 89.8 (84.4-93.4) |  | 451 | 82.5 (76.6-87.2) |  | 403 | 79.0 (73.3-83.8) |  | 407 | 80.8 (76.0-84.8) |  |
| **Country of birth** |  |  |  |  |  |  |  |  |  |  |  |  |
| Australia | 1858 | 81.6 (78.9-84.0) | <0.001 | 1728 | 77.8 (75.4-80.0) | <0.001 | 1723 | 76 (73.6-78.2) | <0.001 | 1527 | 71.4 (68.2-74.5) | 0.001 |
| UK or Ireland | 242 | 90.5 (86.0-93.7) |  | 245 | 85.8 (81.2-89.4) |  | 279 | 81.8 (77.5-85.5) |  | 232 | 79.1 (71.3-85.2) |  |
| Europe | 135 | 91.1 (85.4-94.7) |  | 141 | 89.4 (83.3-93.4) |  | 113 | 92.6 (86.2-96.1) |  | 125 | 85.3 (80.8-88.9) |  |
| Asia | 110 | 70.2 (60.1-78.7) |  | 160 | 67.1 (59.3-74.2) |  | 115 | 58.3 (48.5-67.5) |  | 131 | 63.6 (50.1-75.3) |  |
| Other | 157 | 80.1 (71.5-86.6) |  | 95 | 73.6 (63.3-81.8) |  | 87 | 68.0 (57.4-77.0) |  | 70 | 56.0 (43.0-68.2) |  |
| **Aboriginal / Torres Strait Islander** |  |  |  |  |  |  |  |  |  |  |  |  |
| No | 2472 | 82.7 (80.4-84.7) | <0.001 | 2342 | 78.8 (76.8-80.6) | <0.001 | 2273 | 76.6 (74.6-78.5) | <0.001 | 2040 | 72.3 (69.3-75.1) | <0.001 |
| Yes | 26 | 51.9 (33.5-69.8) |  | 24 | 43.8 (30.0-58.6) |  | 36 | 46.9 (32.2-62.2) |  | 30 | 44.7 (29.7-60.8) |  |
| **Household structure** |  |  |  |  |  |  |  |  |  |  |  |  |
| Couple family children | 928 | 86.9 (83.7-89.5) | <0.001 | 942 | 81.8 (78.7-84.5) | <0.001 | 862 | 78.7 (75.2-81.8) | <0.001 | 818 | 77.9 (73.8-81.6) | <0.001 |
| One parent family, other | 266 | 76.6 (71.0-81.4) |  | 203 | 68.8 (62.3-74.6) |  | 207 | 69.6 (63.9-74.7) |  | 196 | 58.7 (52.5-64.6) |  |
| Lone adult person | 275 | 76.6 (72.8-79.9) |  | 267 | 72.5 (68.6-76.1) |  | 259 | 72.3 (66.5-77.3) |  | 211 | 66.7 (61.7-71.3) |  |
| Couple with no children | 698 | 88.7 (85.9-91.0) |  | 673 | 84.1 (81.2-86.6) |  | 709 | 83.0 (79.9-85.6) |  | 569 | 80.2 (77.2-83.0) |  |
| Other | 335 | 69.3 (63.3-74.6) |  | 284 | 68.2 (62.1-73.7) |  | 279 | 62.2 (55.5-68.4) |  | 291 | 58.3 (52.2-64.1) |  |
| **Marital status** |  |  |  |  |  |  |  |  |  |  |  |  |
| Married/defacto | 1651 | 86.6 (84.5-88.4) | <0.001 | 1532 | 81.9 (79.6-84.0) | <0.001 | 1513 | 79.4 (77.0-81.6) | <0.001 | 1369 | 76.4 (72.9-79.6) | <0.001 |
| Separated/Divorced | 166 | 76.5 (71.2-81.0) |  | 170 | 69.2 (63.7-74.2) |  | 172 | 65.7 (59.9-71.1) |  | 170 | 64.5 (59.9-68.9) |  |
| Widowed | 169 | 93.9 (89.4-96.6) |  | 148 | 92.1 (87.7-95.0) |  | 162 | 91.7 (87.7-94.4) |  | 116 | 87.3 (82.8-90.8) |  |
| Never married | 515 | 70.0 (64.7-74.7) |  | 517 | 68.8 (64.1-73.1) |  | 468 | 65.9 (60.8-70.6) |  | 429 | 59.8 (54.6-64.7) |  |
| **Educational attainment** |  |  |  |  |  |  |  |  |  |  |  |  |
| Secondary schooling | 1153 | 83.2 (79.8-86.2) | 0.148 | 975 | 78.2 (74.8-81.3) | 0.863 | 955 | 76.3 (72.8-79.5) | 0.86 | 849 | 71.5 (66.7-75.9) | 0.869 |
| Trade, certificate, diploma | 834 | 81.2 (78.3-83.7) |  | 914 | 78.6 (76.0-81.1) |  | 844 | 75.4 (72.5-78.1) |  | 761 | 71.8 (68.0-75.2) |  |
| Bachelor degree or higher | 514 | 81.7 (78.2-84.7) |  | 476 | 77.1 (73.2-80.6) |  | 515 | 75.5 (71.6-79.0) |  | 469 | 72.0 (67.1-76.4) |  |
| **Gross annual household income** |  |  |  |  |  |  |  |  |  |  |  |  |
| Up to $20,000 | 227 | 80.9 (74.1-86.3) | 0.134 | 223 | 81.4 (76.2-85.8) | 0.065 | 177 | 74.1 (68.4-79.0) | 0.293 | 120 | 68.3 (61.0-74.8) | 0.006 |
| $20,001 - $40,000 | 336 | 82.0 (77.6-85.7) |  | 321 | 80.0 (75.3-84.0) |  | 257 | 74.8 (69.6-79.3) |  | 290 | 77.8 (71.8-82.8) |  |
| $40,001 - $80,000 | 491 | 80.9 (76.8-84.4) |  | 433 | 73.5 (69.3-77.2) |  | 422 | 72.4 (68.2-76.2) |  | 369 | 65.6 (60.8-70.2) |  |
| $80,001 - $120,000 | 398 | 79.0 (74.5-82.8) |  | 363 | 75.5 (70.7-79.7) |  | 312 | 75.7 (70.7-80.1) |  | 310 | 70.9 (65.3-75.9) |  |
| $120,001 or more | 380 | 86.9 (82.9-90.2) |  | 366 | 80.9 (76.2-84.9) |  | 415 | 78.3 (73.9-82.2) |  | 431 | 73.8 (68.7-78.4) |  |
| Not stated | 671 | 83.1 (78.8-86.6) |  | 663 | 79.5 (74.6-83.6) |  | 734 | 77.4 (73.5-80.9) |  | 565 | 72.8 (68.4-76.8) |  |
| **Employment status** |  |  |  |  |  |  |  |  |  |  |  |  |
| Fulltime employed | 880 | 77.8 (74.6-80.6) | <0.001 | 856 | 73.1 (69.9-76.0) | <0.001 | 803 | 72.0 (68.8-75.0) | <0.001 | 646 | 63.0 (57.3-68.4) | <0.001 |
| Parttime employed | 478 | 81.6 (77.1-85.4) |  | 451 | 77.5 (73.3-81.3) |  | 418 | 75.5 (71.1-79.5) |  | 432 | 73.1 (68.2-77.5) |  |
| Home Duties | 156 | 76.7 (70.1-82.2) |  | 113 | 67.3 (59.1-74.6) |  | 152 | 72.4 (65.7-78.2) |  | 105 | 65.2 (57.2-72.4) |  |
| Unemployed | 41 | 63.1 (48.4-75.8) |  | 58 | 64.0 (51.2-75.1) |  | 37 | 41.5 (30.1-54.0) |  | 55 | 57.4 (42.3-71.2) |  |
| Retired | 585 | 96.3 (94.3-97.6) |  | 574 | 94.0 (91.8-95.7) |  | 576 | 93.9 (92.1-95.4) |  | 545 | 92.0 (89.2-94.1) |  |
| Student | 245 | 83.7 (76.0-89.3) |  | 190 | 78.9 (70.1-85.7) |  | 230 | 69.8 (62.1-76.5) |  | 192 | 69.8 (62.5-76.3) |  |
| Other/Not working due to health | 118 | 73.7 (65.4-80.5) |  | 125 | 76.0 (70.0-81.2) |  | 100 | 69.4 (61.0-76.7) |  | 108 | 66.2 (57.4-74.1) |  |
| **SEIFA IRSD quintile** |  |  |  |  |  |  |  |  |  |  |  |  |
| Lowest (most disadvantaged) | 540 | 75.4 (69.0-80.9) | 0.001 | 523 | 72.5 (68.3-76.4) | <0.001 | 518 | 69.0 (64.9-72.9) | <0.001 | 425 | 64.1 (57.6-70.1) | 0.002 |
| Low | 396 | 81.6 (78.3-84.5) |  | 429 | 70.4 (65.1-75.2) |  | 375 | 73.1 (68.0-77.7) |  | 409 | 68.2 (62.3-73.5) |  |
| Middle | 512 | 82.4 (77.7-86.2) |  | 421 | 79.1 (74.8-82.9) |  | 410 | 74.5 (70.1-78.4) |  | 363 | 71.5 (64.0-78.1) |  |
| High | 474 | 84.9 (80.9-88.2) |  | 432 | 84.2 (80.5-87.2) |  | 444 | 80.0 (75.9-83.6) |  | 420 | 77.4 (70.7-82.9) |  |
| Highest (least disadvantaged) | 580 | 87.4 (83.9-90.2) |  | 564 | 85.9 (82.7-88.6) |  | 569 | 82.9 (79.4-85.9) |  | 468 | 78.6 (74.6-82.2) |  |
| **Dwelling status** |  |  |  |  |  |  |  |  |  |  |  |  |
| Owned or being purchased |  |  |  |  |  |  |  |  |  | 1758 | 81.3 (78.9-83.5) | <0.001 |
| Rent from state government (public housing) |  |  |  |  |  |  |  |  |  | 76 | 54.1 (44.6-63.3) |  |
| Rent privately |  |  |  |  |  |  |  |  |  | 227 | 40.3 (34.6-46.3) |  |
| Other |  |  |  |  |  |  |  |  |  | 16 | 63.3 (37.1-83.4) |  |
| Overall | 2503 | 82.2 (79.9-84.2) |  | 2369 | 78.1 (76.0-80.1) |  | 2316 | 75.8 (73.7-77.8) |  | 2085 | 71.7 (68.5-74.6) |  |

Table S3: Proportion of respondents living in households with at least one mobile telephone by socio-demographic variables, 15 years and over

|  |  | **2010** |  |  | **2011** |  |  | **2012** |  |  | **2013** |  |
| --- | --- | --- | --- | --- | --- | --- | --- | --- | --- | --- | --- | --- |
|  |  | **% (95% CI)** | **p value** |  | **% (95% CI)** | **p value** |  | **% (95% CI)** | **p value** |  | **% (95% CI)** | **p value** |
| **Sex** |  |  |  |  |  |  |  |  |  |  |  |  |
| Male | 1420 | 95.1 (93.6-96.2) | 0.050 | 1440 | 96.9 (96.0-97.5) | <0.001 | 1450 | 97.1 (96.2-97.8) | 0.007 | 1381 | 97.1 (96.2-97.9) | 0.008 |
| Female | 1454 | 93.6 (92.3-94.7) |  | 1435 | 92.9 (91.6-93.9) |  | 1494 | 95.7 (94.5-96.6) |  | 1420 | 95.5 (94.4-96.5) |  |
| **Age (years)** |  |  |  |  |  |  |  |  |  |  |  |  |
| 15 to 24 | 502 | 98.9 (95.8-99.7) | <0.001 | 502 | 99.5 (97.8-99.9) | <0.001 | 483 | 99.2 (96.7-99.8) | <0.001 | 460 | 99.2 (97.0-99.8) | <0.001 |
| 25 to 34 | 464 | 97.5 (92.6-99.2) |  | 473 | 99.9 (99.4-100.0) |  | 471 | 99.8 (99.4-100.0) |  | 443 | 98.6 (96.6-99.4) |  |
| 35 to 44 | 512 | 99.7 (98.8-99.9) |  | 508 | 99.5 (98.6-99.8) |  | 503 | 99.6 (98.2-99.9) |  | 478 | 99.4 (98.3-99.8) |  |
| 45 to 54 | 517 | 98.5 (97.1-99.3) |  | 513 | 98.2 (96.9-98.9) |  | 516 | 98.3 (97.0-99.1) |  | 493 | 98.7 (97.3-99.4) |  |
| 55 to 64 | 435 | 96.2 (94.2-97.6) |  | 430 | 95.5 (93.4-97.0) |  | 454 | 97.6 (96.1-98.5) |  | 434 | 98.0 (96.4-98.9) |  |
| 65 to 74 | 288 | 90.5 (87.2-93.0) |  | 289 | 88.4 (85.1-91.2) |  | 344 | 95.2 (92.9-96.8) |  | 330 | 95.3 (93.6-96.6) |  |
| 75+ | 156 | 61.3 (55.9-66.4) |  | 159 | 65.5 (60.3-70.3) |  | 172 | 71.9 (67.1-76.3) |  | 163 | 72.3 (66.9-77.2) |  |
| **Area of residence** |  |  |  |  |  |  |  |  |  |  |  |  |
| Metropolitan | 2126 | 94.7 (93.8-95.5) | 0.304 | 2134 | 95.4 (94.5-96.2) | 0.003 | 2188 | 96.7 (96.0-97.3) | 0.127 | 2088 | 96.5 (95.5-97.3) | 0.270 |
| Regional | 747 | 93.2 (89.5-95.7) |  | 741 | 93.1 (91.6-94.4) |  | 756 | 95.3 (92.8-97.0) |  | 713 | 95.7 (94.5-96.7) |  |
| **Number of people in household** |  |  |  |  |  |  |  |  |  |  |  |  |
| 1 | 313 | 77.3 (74.1-80.2) | <0.001 | 334 | 79.5 (76.8-82.0) | <0.001 | 342 | 84.0 (81.3-86.4) | <0.001 | 320 | 85.3 (81.8-88.2) | <0.001 |
| 2 | 1527 | 95.6 (94.2-96.7) |  | 1497 | 95.9 (94.8-96.8) |  | 1560 | 97.7 (96.9-98.3) |  | 1473 | 97.3 (96.4-97.9) |  |
| 3 | 571 | 98.1 (96.0-99.1) |  | 498 | 98.9 (97.0-99.6) |  | 533 | 98.5 (96.7-99.3) |  | 508 | 98.8 (96.9-99.6) |  |
| 4 or more | 462 | 100.0 |  | 545 | 99.7 (98.0-100.0) |  | 509 | 99.7 (98.0-100.0) |  | 500 | 99.1 (96.6-99.8) |  |
| **Country of birth** |  |  |  |  |  |  |  |  |  |  |  |  |
| Australia | 2164 | 95.0 (93.7-96.1) | <0.001 | 2126 | 95.7 (94.9-96.4) | <0.001 | 2195 | 96.8 (95.9-97.5) | <0.001 | 2068 | 96.7 (96.0-97.4) | <0.001 |
| UK or Ireland | 240 | 89.9 (86.5-92.6) |  | 258 | 90.4 (86.9-93.0) |  | 325 | 95.2 (93.1-96.6) |  | 281 | 95.7 (93.2-97.4) |  |
| Europe | 128 | 86.0 (80.6-90.1) |  | 134 | 85.3 (79.3-89.8) |  | 108 | 89.1 (82.8-93.3) |  | 130 | 88.4 (81.5-93.0) |  |
| Asia | 156 | 99.1 (96.3-99.8) |  | 236 | 98.9 (96.6-99.6) |  | 193 | 97.9 (94.5-99.2) |  | 204 | 99.3 (96.3-99.9) |  |
| Other | 186 | 94.7 (91.2-96.9) |  | 121 | 93.6 (88.3-96.5) |  | 123 | 96.6 (91.9-98.6) |  | 118 | 95.0 (86.7-98.2) |  |
| **Aboriginal / Torres Strait Islander** |  |  |  |  |  |  |  |  |  |  |  |  |
| No | 2826 | 94.5 (93.4-95.4) | 0.078 | 2819 | 94.8 (94.0-95.5) | 0.778 | 2860 | 96.4 (95.6-97.1) | 0.947 | 2719 | 96.4 (95.5-97.0) | 0.140 |
| Yes | 43 | 84.9 (71.2-92.8) |  | 52 | 96.3 (87.9-98.9) |  | 74 | 95.9 (92.0-98.0) |  | 65 | 97.2 (91.8-99.1) |  |
| **Household structure** |  |  |  |  |  |  |  |  |  |  |  |  |
| Couple family children | 1063 | 99.6 (98.3-99.9) | <0.001 | 1146 | 99.4 (98.6-99.8) | <0.001 | 1090 | 99.5 (98.7-99.8) | <0.001 | 1044 | 99.5 (98.6-99.9) | <0.001 |
| One parent family, other | 344 | 98.8 (97.1-99.5) |  | 293 | 99.3 (97.9-99.8) |  | 295 | 99.1 (96.4-99.8) |  | 332 | 99.4 (98.3-99.8) |  |
| Lone adult person | 267 | 74.3 (70.4-77.8) |  | 282 | 76.6 (73.4-79.4) |  | 294 | 82.2 (79.2-84.8) |  | 259 | 81.7 (77.9-85.0) |  |
| Couple with no children | 743 | 94.3 (92.5-95.7) |  | 751 | 93.8 (92.0-95.2) |  | 824 | 96.3 (94.8-97.4) |  | 680 | 95.8 (94.1-97.0) |  |
| Other | 457 | 94.5 (91.9-96.3) |  | 403 | 97.0 (94.9-98.3) |  | 441 | 98.3 (96.5-99.2) |  | 486 | 97.5 (96.0-98.5) |  |
| **Marital status** |  |  |  |  |  |  |  |  |  |  |  |  |
| Married/defacto | 1847 | 96.9 (96.0-97.6) | <0.001 | 1809 | 96.7 (95.8-97.4) | <0.001 | 1866 | 97.9 (97.2-98.5) | <0.001 | 1752 | 97.8 (97.1-98.4) | <0.001 |
| Separated/Divorced | 199 | 91.6 (88.3-94.1) |  | 220 | 89.5 (86.5-91.9) |  | 248 | 95.1 (92.9-96.6) |  | 249 | 94.3 (91.5-96.2) |  |
| Widowed | 113 | 62.4 (56.5-68.0) |  | 110 | 68.2 (62.7-73.3) |  | 132 | 75.0 (70.0-79.4) |  | 97 | 73.1 (67.5-78.2) |  |
| Never married | 709 | 96.3 (92.9-98.1) |  | 732 | 97.5 (96.2-98.4) |  | 695 | 98.0 (96.4-98.8) |  | 703 | 97.9 (96.4-98.8) |  |
| **Educational attainment** |  |  |  |  |  |  |  |  |  |  |  |  |
| Secondary schooling | 1256 | 90.7 (88.9-92.1) | <0.001 | 1140 | 91.5 (89.9-92.8) | <0.001 | 1175 | 93.9 (92.3-95.2) | <0.001 | 1113 | 93.8 (92.2-95.1) | <0.001 |
| Trade, certificate, diploma | 990 | 96.4 (95.0-97.4) |  | 1123 | 96.6 (95.6-97.3) |  | 1095 | 97.8 (97.0-98.5) |  | 1037 | 97.8 (96.8-98.5) |  |
| Bachelor degree or higher | 625 | 99.4 (98.6-99.7) |  | 607 | 98.2 (97.1-98.9) |  | 672 | 98.6 (97.5-99.2) |  | 644 | 98.9 (97.5-99.5) |  |
| **Gross annual household income** |  |  |  |  |  |  |  |  |  |  |  |  |
| Up to $20,000 | 194 | 69.3 (64.3-73.9) | <0.001 | 211 | 77.1 (73.1-80.7) | <0.001 | 200 | 83.7 (79.6-87.1) | <0.001 | 137 | 78.3 (73.7-82.3) | <0.001 |
| $20,001 - $40,000 | 378 | 92.3 (89.3-94.5) |  | 361 | 90.2 (87.4-92.4) |  | 327 | 95.1 (92.8-96.7) |  | 345 | 92.6 (89.6-94.7) |  |
| $40,001 - $80,000 | 594 | 97.9 (96.5-98.8) |  | 583 | 98.7 (97.7-99.3) |  | 572 | 98.2 (96.9-99.0) |  | 554 | 98.4 (97.2-99.1) |  |
| $80,001 - $120,000 | 502 | 99.7 (98.6-99.9) |  | 475 | 98.8 (97.2-99.4) |  | 406 | 98.8 (97.3-99.4) |  | 435 | 99.4 (97.6-99.9) |  |
| $120,001 or more | 438 | 100.0 |  | 452 | 99.9 (99.4-100.0) |  | 530 | 100.0 |  | 582 | 99.6 (98.3-99.9) |  |
| Not stated | 767 | 95 (92.9-96.4) |  | 793 | 95.1 (93.5-96.3) |  | 909 | 95.8 (94.0-97.1) |  | 748 | 96.4 (94.6-97.6) |  |
| **Employment status** |  |  |  |  |  |  |  |  |  |  |  |  |
| Fulltime employed | 1122 | 99.2 (98.0-99.7) | <0.001 | 1161 | 99.1 (98.5-99.5) | <0.001 | 1107 | 99.3 (98.6-99.7) | <0.001 | 1016 | 99.1 (98.3-99.5) | <0.001 |
| Parttime employed | 582 | 99.4 (97.7-99.8) |  | 573 | 98.4 (97.2-99.1) |  | 549 | 99.2 (98.2-99.7) |  | 586 | 99.2 (98.1-99.7) |  |
| Home Duties | 192 | 94.4 (90.7-96.7) |  | 166 | 99.0 (96.7-99.7) |  | 203 | 96.6 (93.1-98.3) |  | 158 | 98.2 (95.2-99.3) |  |
| Unemployed | 65 | 99.0 (93.2-99.9) |  | 88 | 98.0 (93.6-99.4) |  | 88 | 98.5 (94.2-99.6) |  | 95 | 99.0 (95.8-99.8) |  |
| Retired | 482 | 79.3 (76.1-82.3) |  | 486 | 79.7 (76.8-82.3) |  | 536 | 87.3 (84.8-89.5) |  | 514 | 86.7 (84.2-88.9) |  |
| Student | 287 | 98.4 (94.1-99.6) |  | 239 | 99.3 (95.1-99.9) |  | 328 | 99.4 (96.0-99.9) |  | 273 | 99.1 (95.8-99.8) |  |
| Other/not working due to health | 143 | 89.0 (81.8-93.5) |  | 155 | 94.7 (91.4-96.8) |  | 133 | 92.6 (88.9-95.2) |  | 157 | 96.6 (93.3-98.3) |  |
| **SEIFA IRSD quintile** |  |  |  |  |  |  |  |  |  |  |  |  |
| Lowest (most disadvantaged) | 657 | 91.8 (88.2-94.5) | 0.001 | 683 | 94.8 (93.3-95.9) | 0.015 | 714 | 95.2 (92.8-96.8) | 0.113 | 630 | 95.1 (93.0-96.5) | 0.015 |
| Low | 453 | 93.3 (90.6-95.2) |  | 565 | 92.8 (90.5-94.6) |  | 495 | 96.5 (94.8-97.6) |  | 572 | 95.4 (93.9-96.5) |  |
| Middle | 588 | 94.6 (92.7-96.0) |  | 506 | 95.0 (93.0-96.5) |  | 529 | 96.0 (94.4-97.1) |  | 490 | 96.5 (94.8-97.7) |  |
| High | 526 | 94.3 (92.3-95.8) |  | 485 | 94.6 (92.7-96.0) |  | 536 | 96.6 (94.8-97.8) |  | 525 | 96.7 (95.2-97.8) |  |
| Highest (least disadvantaged) | 648 | 97.6 (96.4-98.4) |  | 636 | 96.8 (95.1-97.9) |  | 671 | 97.7 (96.5-98.5) |  | 584 | 98.2 (96.5-99.1) |  |
| **Dwelling status** |  |  |  |  |  |  |  |  |  |  |  |  |
| Owned or being purchased |  |  |  |  |  |  |  |  |  | 2082 | 96.3 (95.3-97.1) | <0.001 |
| Rent from state government (public housing) |  |  |  |  |  |  |  |  |  | 128 | 91.2 (87.2-94.1) |  |
| Rent privately |  |  |  |  |  |  |  |  |  | 553 | 98.3 (96.9-99.1) |  |
| Other |  |  |  |  |  |  |  |  |  | 23 | 89.9 (73.1-96.7) |  |
| Overall | 2873 | 94.3 (93.2-95.3) |  | 2875 | 94.8 (94.1-95.5) |  | 2944 | 96.4 (95.6-97.0) |  | 2801 | 96.3 (95.5-97.0) |  |

Table S4: Proportion of respondents living in households with a directory-listed telephone number (EWP) by socio-demographic variables, 15 years and over

|  |  | **2010** |  |  | **2011** |  |  | **2012** |  |  | **2013** |  |
| --- | --- | --- | --- | --- | --- | --- | --- | --- | --- | --- | --- | --- |
|  |  | **% (95% CI)** | **p value** |  | **% (95% CI)** | **p value** |  | **% (95% CI)** | **p value** |  | **% (95% CI)** | **p value** |
| **Sex** |  |  |  |  |  |  |  |  |  |  |  |  |
| Male | 878 | 59.0 (55.9-62.0) | 0.153 | 819 | 55.2 (51.9-58.5) | 0.55 | 761 | 51.0 (47.9-54.2) | 0.099 | 716 | 50.5 (46.0-55.0) | 0.439 |
| Female | 958 | 61.8 (58.8-64.7) |  | 872 | 56.4 (53.4-59.4) |  | 852 | 54.6 (51.6-57.5) |  | 724 | 48.7 (45.0-52.5) |  |
| **Age (years)** |  |  |  |  |  |  |  |  |  |  |  |  |
| 15 to 24 | 216 | 42.6 (36.6-48.8) | <0.001 | 200 | 39.7 (33.5-46.2) | <0.001 | 176 | 36.2 (30.5-42.2) | <0.001 | 141 | 30.4 (23.7-38.0) | <0.001 |
| 25 to 34 | 189 | 39.9 (34.7-45.4) |  | 149 | 31.5 (26.4-37.1) |  | 141 | 30.0 (25.2-35.1) |  | 85 | 18.9 (14.7-24.0) |  |
| 35 to 44 | 301 | 58.6 (53.6-63.5) |  | 232 | 45.4 (40.5-50.4) |  | 219 | 43.4 (38.5-48.5) |  | 208 | 43.4 (38.3-48.6) |  |
| 45 to 54 | 324 | 61.8 (57.2-66.2) |  | 326 | 62.5 (57.3-67.5) |  | 299 | 57.1 (51.8-62.1) |  | 277 | 55.4 (49.8-60.9) |  |
| 55 to 64 | 334 | 74.2 (69.8-78.1) |  | 326 | 72.5 (68.2-76.5) |  | 292 | 62.8 (58.6-66.9) |  | 289 | 65.2 (60.8-69.3) |  |
| 65 to 74 | 266 | 83.9 (80.0-87.2) |  | 252 | 77.0 (72.2-81.2) |  | 299 | 82.7 (79.1-85.8) |  | 255 | 73.8 (68.8-78.3) |  |
| 75+ | 206 | 81.3 (76.3-85.4) |  | 206 | 85.1 (80.6-88.8) |  | 187 | 78.2 (72.1-83.3) |  | 186 | 82.6 (76.7-87.3) |  |
| **Area of residence** |  |  |  |  |  |  |  |  |  |  |  |  |
| Metropolitan | 1331 | 59.3 (57.1-61.5) | 0.229 | 1216 | 54.4 (51.8-56.9) | 0.108 | 1191 | 52.7 (50.1-55.2) | 0.793 | 1047 | 48.5 (44.4-52.6) | 0.254 |
| Regional | 505 | 63.6 (56.9-69.8) |  | 476 | 60.0 (53.6-66.1) |  | 423 | 53.3 (49.1-57.6) |  | 393 | 52.9 (46.3-59.3) |  |
| **Number of people in household** |  |  |  |  |  |  |  |  |  |  |  |  |
| 1 | 234 | 58.6 (54.8-62.3) | 0.118 | 218 | 51.9 (48.0-55.9) | 0.545 | 210 | 51.9 (46.7-57.0) | 0.931 | 176 | 47.3 (42.2-52.4) | 0.248 |
| 2 | 1005 | 63.0 (59.9-66.0) |  | 880 | 56.4 (53.6-59.2) |  | 853 | 53.5 (50.9-56.0) |  | 765 | 50.5 (46.7-54.2) |  |
| 3 | 331 | 57.1 (51.9-62.0) |  | 283 | 56.2 (50.3-61.9) |  | 286 | 52.8 (47.0-58.6) |  | 237 | 46.4 (41.1-51.7) |  |
| 4 or more | 265 | 57.4 (50.2-64.3) |  | 311 | 56.9 (50.1-63.5) |  | 264 | 51.7 (45.0-58.4) |  | 262 | 52.0 (45.4-58.5) |  |
| **Country of birth** |  |  |  |  |  |  |  |  |  |  |  |  |
| Australia | 1402 | 61.8 (59.1-64.5) | <0.001 | 1255 | 56.6 (53.7-59.4) | <0.001 | 1217 | 53.7 (51.3-56.1) | <0.001 | 1108 | 51.9 (47.7-56.1) | <0.001 |
| UK or Ireland | 184 | 68.9 (63.0-74.3) |  | 175 | 61.4 (55.3-67.2) |  | 181 | 53.2 (47.0-59.3) |  | 148 | 50.5 (44.4-56.5) |  |
| Europe | 92 | 61.7 (53.6-69.3) |  | 101 | 64.3 (56.9-71.1) |  | 77 | 63.5 (54.9-71.3) |  | 88 | 59.7 (51.7-67.3) |  |
| Asia | 56 | 35.8 (28.0-44.5) |  | 91 | 38.0 (30.8-45.7) |  | 65 | 33.2 (24.9-42.6) |  | 56 | 27.0 (20.8-34.2) |  |
| Other | 101 | 51.6 (42.4-60.7) |  | 70 | 53.6 (43.9-63.1) |  | 72 | 56.5 (46.0-66.4) |  | 41 | 33.1 (24.0-43.8) |  |
| **Aboriginal / Torres Strait Islander** |  |  |  |  |  |  |  |  |  |  |  |  |
| No | 1815 | 60.8 (58.4-63.0) | 0.018 | 1674 | 56.3 (53.9-58.7) | 0.006 | 1585 | 53.5 (51.3-55.6) | 0.001 | 1420 | 50.4 (46.9-53.9) | <0.001 |
| Yes | 16 | 35.9 (22.3-52.1) |  | 16 | 30.0 (16.1-48.9) |  | 21 | 27.8 (17.9-40.6) |  | 11 | 15.9 (9.1-26.3) |  |
| **Household structure** |  |  |  |  |  |  |  |  |  |  |  |  |
| Couple family children | 685 | 64.1 (60.2-67.8) | <0.001 | 644 | 55.9 (51.8-59.9) | <0.001 | 584 | 53.4 (49.4-57.3) | <0.001 | 538 | 51.3 (46.6-56.0) | <0.001 |
| One parent family, other | 162 | 46.8 (41.0-52.7) |  | 109 | 37.0 (30.6-43.8) |  | 121 | 40.7 (35.1-46.7) |  | 105 | 31.4 (26.3-37.0) |  |
| Lone adult person | 214 | 60.3 (56.1-64.4) |  | 200 | 54.6 (50.5-58.7) |  | 196 | 55.1 (49.8-60.3) |  | 160 | 51.0 (46.2-55.8) |  |
| Couple with no children | 550 | 69.8 (66.7-72.8) |  | 537 | 67.2 (63.5-70.6) |  | 535 | 62.6 (59.4-65.6) |  | 446 | 62.9 (58.6-67.1) |  |
| Other | 225 | 46.8 (40.9-52.9) |  | 201 | 48.3 (41.9-54.7) |  | 176 | 39.3 (33.1-45.9) |  | 191 | 38.4 (32.3-44.9) |  |
| **Marital status** |  |  |  |  |  |  |  |  |  |  |  |  |
| Married/defacto | 1261 | 66.2 (63.8-68.5) | <0.001 | 1127 | 60.3 (57.5-63.0) | <0.001 | 1101 | 57.8 (55.2-60.3) | <0.001 | 998 | 55.7 (51.9-59.5) | <0.001 |
| Separated/Divorced | 126 | 58.4 (52.0-64.6) |  | 125 | 51.0 (45.1-56.9) |  | 112 | 43.1 (37.9-48.4) |  | 126 | 47.7 (42.9-52.5) |  |
| Widowed | 130 | 72.2 (65.7-77.8) |  | 120 | 74.6 (68.7-79.7) |  | 129 | 73.2 (67.4-78.3) |  | 91 | 68.7 (57.9-77.9) |  |
| Never married | 319 | 43.6 (38.6-48.7) |  | 319 | 42.5 (37.6-47.6) |  | 269 | 38.0 (33.2-43.0) |  | 226 | 31.6 (26.6-37.0) |  |
| **Educational attainment** |  |  |  |  |  |  |  |  |  |  |  |  |
| Secondary schooling | 820 | 59.5 (56.2-62.8) | 0.364 | 693 | 55.6 (52.0-59.2) | 0.341 | 662 | 53.0 (49.8-56.1) | 0.468 | 568 | 48.0 (42.6-53.4) | 0.23 |
| Trade, certificate, diploma | 638 | 62.1 (58.6-65.4) |  | 670 | 57.7 (54.0-61.2) |  | 597 | 53.4 (50.0-56.8) |  | 557 | 52.6 (49.1-56.0) |  |
| Bachelor degree or higher | 377 | 59.9 (55.8-63.9) |  | 326 | 52.9 (48.3-57.4) |  | 354 | 51.9 (47.7-56.1) |  | 311 | 47.7 (41.9-53.6) |  |
| **Gross annual household income** |  |  |  |  |  |  |  |  |  |  |  |  |
| Up to $20,000 | 169 | 62.0 (55.3-68.3) | 0.398 | 170 | 62.7 (56.8-68.2) | 0.103 | 130 | 54.8 (49.5-60.0) | 0.367 | 86 | 49.6 (43.0-56.1) | 0.002 |
| $20,001 - $40,000 | 257 | 62.9 (57.1-68.3) |  | 244 | 61.0 (55.6-66.1) |  | 200 | 58.3 (53.3-63.1) |  | 229 | 61.5 (56.3-66.5) |  |
| $40,001 - $80,000 | 366 | 60.3 (56.0-64.5) |  | 313 | 53.1 (48.1-58.0) |  | 306 | 52.6 (48.1-57.0) |  | 266 | 47.4 (42.5-52.3) |  |
| $80,001 - $120,000 | 301 | 59.8 (55.1-64.2) |  | 258 | 53.7 (48.0-59.2) |  | 207 | 50.4 (44.9-55.8) |  | 223 | 51.0 (44.8-57.2) |  |
| $120,001 or more | 278 | 63.6 (58.6-68.3) |  | 253 | 55.8 (50.4-61.1) |  | 279 | 52.7 (47.6-57.6) |  | 279 | 47.7 (42.0-53.4) |  |
| Not stated | 464 | 57.4 (53.0-61.8) |  | 454 | 54.4 (49.4-59.3) |  | 491 | 51.8 (47.7-55.8) |  | 357 | 46.2 (40.2-52.3) |  |
| **Employment status** |  |  |  |  |  |  |  |  |  |  |  |  |
| Fulltime employed | 649 | 57.4 (54.1-60.6) | <0.001 | 586 | 50.0 (46.3-53.7) | <0.001 | 547 | 49.1 (45.4-52.7) | <0.001 | 442 | 43.1 (38.0-48.5) | <0.001 |
| Parttime employed | 353 | 60.3 (55.7-64.7) |  | 314 | 53.9 (48.7-59.1) |  | 262 | 47.4 (42.4-52.4) |  | 301 | 50.9 (46.5-55.4) |  |
| Home Duties | 101 | 49.8 (42.4-57.3) |  | 64 | 38.4 (31.3-45.9) |  | 106 | 50.2 (42.0-58.3) |  | 72 | 44.9 (38.0-52.1) |  |
| Unemployed | 25 | 37.9 (25.7-51.8) |  | 37 | 41.1 (29.9-53.4) |  | 27 | 31.1 (20.5-44.2) |  | 34 | 36.0 (22.9-51.5) |  |
| Retired | 485 | 80.0 (76.6-82.9) |  | 483 | 79.3 (76.0-82.3) |  | 471 | 76.9 (73.7-79.9) |  | 447 | 75.6 (71.3-79.4) |  |
| Student | 146 | 50.1 (42.1-58.1) |  | 111 | 46.2 (36.6-56.1) |  | 134 | 40.7 (33.7-48.2) |  | 81 | 29.3 (21.6-38.4) |  |
| Other/not working due to health | 77 | 49.7 (39.3-60.0) |  | 93 | 57.2 (49.1-64.9) |  | 65 | 45.8 (38.4-53.4) |  | 62 | 38.7 (31.7-46.2) |  |
| **SEIFA IRSD quintile** |  |  | 0.01 |  |  |  |  |  |  |  |  |  |
| Lowest (most disadvantaged) | 378 | 53.4 (47.6-59.1) |  | 369 | 51.2 (45.4-57.0) | <0.001 | 379 | 50.7 (46.6-54.7) | 0.162 | 289 | 43.7 (37.6-50.0) | 0.072 |
| Low | 298 | 61.5 (57.4-65.5) |  | 297 | 48.9 (44.3-53.5) |  | 273 | 53.4 (47.8-58.8) |  | 279 | 46.5 (41.9-51.2) |  |
| Middle | 385 | 62 (57.4-66.5) |  | 304 | 57.2 (51.3-62.9) |  | 274 | 49.8 (45.1-54.5) |  | 263 | 51.8 (44.0-59.6) |  |
| High | 348 | 62.3 (57.2-67.1) |  | 302 | 58.8 (53.7-63.8) |  | 291 | 52.5 (47.3-57.7) |  | 284 | 52.3 (44.7-59.8) |  |
| Highest (least disadvantaged) | 426 | 64.1 (60.4-67.7) |  | 420 | 63.9 (59.2-68.4) |  | 395 | 57.6 (52.7-62.2) |  | 327 | 54.9 (50.0-59.8) |  |
| **Dwelling status** |  |  |  |  |  |  |  |  |  |  |  |  |
| Owned or being purchased |  |  |  |  |  |  |  |  |  | 1276 | 59.0 (55.4-62.6) | <0.001 |
| Rent from state government (public housing) |  |  |  |  |  |  |  |  |  | 46 | 33.6 (25.6-42.6) |  |
| Rent privately |  |  |  |  |  |  |  |  |  | 105 | 18.7 (15.1-23.0) |  |
| Other |  |  |  |  |  |  |  |  |  | 11 | 43.1 (25.7-62.5) |  |
| Overall | 1836 | 60.4 (58.1-62.7) |  | 1692 | 55.8 (53.4-58.2) |  | 1613 | 52.9 (50.7-55.0) |  | 1440 | 49.6 (46.1-53.1) |  |
